# Relieving Sore Throat Formula Exerts a Therapeutic Effect on Pharyngitis through Immunoregulation and NF-*κ*B Pathway

**DOI:** 10.1155/2020/2929163

**Published:** 2020-05-15

**Authors:** Yushi Ding, Suyun Yu, Zhonghong Wei, Rui Deng, Peng Chen, Yifan Sun, Qi Jia, Xiaoman Li, Yuanyuan Wu, Wenxing Chen, Kurt S. Zanker, Aiyun Wang, Yin Lu

**Affiliations:** ^1^Jiangsu Key Laboratory for Pharmacology and Safety Evaluation of Chinese Materia Medica, School of Pharmacy, Nanjing University of Chinese Medicine, Nanjing 210023, China; ^2^Jiangsu Collaborative Innovation Center of Traditional Chinese Medicine Prevention and Treatment of Tumor, Nanjing University of Chinese Medicine, Nanjing 210023, China; ^3^Institute for Immunology, Witten/Herdecke University, Witten, Germany

## Abstract

Relieving Sore Throat Formula (RSTF) is a formula approved by the China Food and Drug Administration and has been used for the treatment of pharyngitis in clinic for many years. However, the potential pharmacological mechanism still remains unknown. We combined multiple methods including bioinformatics data digging, network pharmacology analysis, and pathway analysis to predict the potential target of RSTF. We verified our in silico prediction results with an in vivo/vitro antibacterial effect test, mouse phagocytic index test, proliferation, transformation, and migration of mouse spleen lymphocytes. Alteration of NF-*κ*B pathway was determined by Western blotting, immunofluorescence, and PCR. The in vivo experiments demonstrated that the RSTF could significantly relieve the symptoms of pharyngitis. A rat saliva secretion test showed that RSTF can effectively relieve the xerostomia symptom. A phenol red excretion test showed that RSTF has an eliminating phlegm effect. A hot plate method and granuloma experiment proved that RSTF also have analgesic and anti-inflammatory effects. In silico prediction demonstrates that 70 active compounds of RSTF were filtered out through ADME screening and 84 putative targets correlated with different diseases. Pathway enrichment analysis showed that the candidate targets were mostly related to the response to bacteria and immunity signalling pathways, which are known contributors to pharyngitis. Experimental results confirmed that RSTF exerted therapeutic effects on pharyngitis mainly by antibacterial effect and downregulation of NF-*κ*B activities. It is demonstrated both in silico and in vivo/vitro that RSTF exerted therapeutic effects on pharyngitis mainly through an antibiotic effect and downregulation of NF-*κ*B signalling pathway.

## 1. Introduction

Pharyngitis is the inflammation with both various infectious and noninfectious causes in the pharyngeal mucosa, mucous membrane, or lymphoid tissue and is part of the upper respiratory tract inflammation [1]. Pharyngitis is divided into acute and chronic types. Most of acute pharyngitis are caused by infection, and chronic pharyngitis tends to be secondary to acute pharyngitis except for other external factors, which can be complicated by some downward inflammation, such as acute or chronic laryngitis, bronchitis, and pneumonia [1, 2]. The deterioration of infection may also lead to systemic complications such as acute nephritis, sepsis, rheumatic fever, and other diseases [3–5]. The incidence of pharyngitis has reached 20%-50% according to the epidemiological statistics [6, 7]. Pharyngitis is a common upper respiratory tract disease in adults with long course of disease and stubborn symptom which are difficult to cure. Most patients suffer from a variety of pharyngeal discomforts, such as foreign body sensation, burning sensation, dryness, itching, slight pain, and frequent irritative cough in the morning, and some even have nausea and vomit symptom [8]. It is now considered that pharyngitis is not only a physical disease but also a psychological disorder in many patients [9]. Recurrent pharyngitis is refractory and often caused a great influence on the life and work of patients. Consequently, it is urgent to ease the progression of pharyngitis.

The pathogenesis of pharyngitis remains unclear so far. It is reported that adjacent upper respiratory tract lesions, climate and geographical environment changes, occupational factors, systemic factors, and allergic factors can induce pharyngitis [1]. The occurrence and development of pharyngitis are the results of the interaction of multiple signalling pathways which are composed of a disorder polygenic molecular network. Most medicines used in clinic to treat pharyngitis are antibiotics or a single component drug with limited targets, and the therapeutic effect is often unsatisfactory [10]. After a long period of treatment, the patient will become resistant to the drug, which will even cause severe toxic and side effects [11–13]. Thus, investigators in China and other Asian countries have endeavoured to seek alternative treatments for pharyngitis from TCM or natural herbs, some of which have shown promising effects in clinical studies.

RSTF has been used for the treatment of pharyngitis in clinic for many years, which is comprised of seven herbs, including Hedysarum Multijugum Maxim (HMM), Fructus Ligustri Lucidi (FLL), Atractylodes macrocephala Koidz (AMK), Curcumae Radix (CR), Poria Cocos (Schw.) Wolf (PCW), Oroxyli Semen (OS), Euphorbiae Helioscopiae Herba (EHH). These seven traditional Chinese medicines contain many effective ingredients, which may exert an “added” effect by acting on the same single target or a synergy effect produced between individual targets at the same time; all of these constitute the important connotation of the pharmacodynamic mechanism. TCMs achieve therapeutic effects mainly by targeting multiple physiological pathways at the same time. However, as far as the current research of traditional Chinese medicines is concerned, the active agents in Chinese herbal medicines are numerous, which are also difficult to identify [14]. Although RSTF is effective in treating pharyngitis in clinic, the potential pharmacological mechanism still remains unknown. Therefore, there is an urgency to seek an efficient and accurate method to clarify the mechanism behind.

With the development of the network pharmacology technology, the research idea of TCM transforms from single component-single target to systemic regulation. In 2007, Yildirim et al. first applied the concept of network biology. Through the integration analysis of drug-gene and drug-protein interaction data, it was found that most drugs act through indirect regulation rather than acting directly on disease-related proteins [15]. In the same year, Hopkins proposed a network pharmacology research method. It is believed that drugs act on multiple targets and produce synergism and attenuation effects through interactions between multiple targets. The current combination of omics technologies and bioinformatics provides computational and experimental strategies toward network pharmacology [16–18]. Using a systematic pharmacology approach, we investigated the pharmacological mechanism of RSTF with the goal of understanding its effects at the system, organ, and molecular levels. An in vivo experiment showed that RSTF exert remarkable promoting fluid, moistening dryness, resolving phlegm, and analgesic effects. Meanwhile, both in vivo and in vitro pharmacodynamics experiments indicated that RSTF can relieve the symptoms of pharyngitis by antimicrobial and immune regulatory function, which is consistent with the in silico predictions. Network pharmacology study problems from the perspective of mutual connection exactly coincide with the core ideas of traditional Chinese medicine. Therefore, the application of network pharmacology to TCM has unique advantages and great potential for development.

## 2. Materials and Methods

### 2.1. Reagents and Animals

RSTF is composed of seven plant materials, including HMM, FLL, AMK, CR, PCW, OS, and EHH. Yu-ping-feng particle (YPFP) was purchased from Sinopharm Group Guangdong Global Pharmaceutical Co., Ltd. (China); Lanqin oral liquid was from Yangzi River Pharmaceutical Group Co., Ltd. (China); Dexamethasone acetate (DXM) and lipopolysaccharide were purchased from Sigma-Aldrich (USA); aspirin was from Bayer (Germany); ambroxol hydrochloride and atropine were from Aladdin (China), concanavalin (ConA) was from Biosharp (China); tumor necrosis factor (TNF-*α*) was from Bioss (China). The antibodies of GAPDH, NF-*κ*B, and p65-NF-*κ*B were purchased from Abcam (UK).

All animals were bought from Shanghai SIPPR/BK Experimental Animal Co. They were raised under controlled humidity (55-65%), temperature (22 ± 2°C), and daily light intensity (12 h/12 h light/dark cycles) and fed with water and standard diets ad libitum. Experimental protocols have been approved by the Committee on Laboratory Animal Care of Nanjing University of Chinese Medicine, and all animals were given humane care according to the guidelines of the National Institutes of Health (USA).

### 2.2. Pharmacodynamics

#### 2.2.1. Rat Saliva Secretion Test

Male rats weighing 180 g-220 g were randomly divided into six groups, with intragastric administration (1 mL/100 g) once daily, continuous administration for six days, and fasting for twelve hours before the test; water was not allowed. Atropine (0.16 mg/kg) was given by gavage thirty minutes after the last administration, rats were anaesthetized, and the salivary secretion was measured. The rats were fixed, and the dry cotton balls (50 ± 5 mg) were placed under the tongue of the rats to measure the saliva secretion of the rats. Each cotton ball was changed every thirty minutes for two hours; the maximum saliva secretion was calculated from the weight difference between the cotton balls.

#### 2.2.2. Phenol Red Excretion Test

ICR mice were randomly divided into five groups of ten in each group: blank control group, positive drug group (ambroxol hydrochloride), and three doses of RSTF group (5.625 g/kg, 11.25 g/kg, and 22.5 g/kg). Mice were intragastrically administered once a day for eight consecutive days, and 2.5% phenol red saline solution (0.1 mL/10 g) was administered intraperitoneally at 30 minutes after the last administration. Mice were euthanized and fixed after thirty minutes of injection, and the trachea is cannulated and connected to the syringe. Combine three lavage fluids and place them for a period of time to precipitate impurities; the clear red supernatant was taken, and its absorbance was measured with a spectrophotometer at 545 nm. Calculate the amount of phenol red based on the standard curve.

#### 2.2.3. Sialic Acid Secretion Test

Rats in each group were intragastrically administered at 1 mL/100 g (the control group was given an equal volume of normal saline) once daily for seven consecutive days. Rats were euthanized, and the trachea is cannulated after one hour of the last administration. 1 mL prewarmed saline (37°C) was slowly inhaled into the trachea and then gently sucked out; this was repeated this twice. Take the supernatant after centrifugation, and detect it with a sialic acid (SA) test box within two hours. Each sample was divided into two dilutions, and each dilution was measured twice and averaged.

#### 2.2.4. Hot Plate Method

The temperature of the thermostat hot plate is adjusted to 55 ± 0.5°C, and the plate is placed in a large beaker for fixing. The time between the mouse put into the hot plate and licking rear feet is the pain reaction time. Screened qualified mice with a licking rear feet reaction within 10-30 seconds were subjected to follow-up experiments (escapers and jumpers were abandoned). Once the mice had a licking rear feet reaction, they were immediately taken out to prevent hurts. Fifty qualified mice were selected and randomly divided into five groups of ten for succeeding experiments. The pain threshold of each group of mice prior to administration was measured and recorded. After seven days of continuous administration, pain response was measured at 15, 30, 45, and 60 min after the seventh day of administration. In the test, if mice have no painful reaction within 60 seconds, they should be immediately taken out and the result should be recorded according to 60 seconds. The percentage increase was calculated as follows: percentage increase in pain threshold = (postdrug pain reaction time − predrug pain reaction time)/predrug pain reaction time × 100%.

#### 2.2.5. Model of Cotton Ball Granuloma in Rats

Male rats weighing 180 g-220 g were anaesthetized and randomly divided into five groups; abdominal hair was removed carefully. Two sterilized cotton balls (each weighing 50 ± 5 mg, autoclaved, containing 1 mg/0.1 mL of ampicillin, and oven-dried at 50°C) were, respectively, implanted into the both sides of inguinal subcutaneous ring of rats; suture the wound with a surgical line. In continuous administration for seven days, rats were euthanized on the eighth day. The cotton balls were taken out and weighed after being dried twelve hours at 60°C; subtract the weight of the original cotton ball as the weight of granuloma.

#### 2.2.6. Agar-Induced Rat Granuloma Model

The rats were anaesthetized with isoflurane and operated under aseptic conditions, and 2 mL of a 2% agar solution was injected subcutaneously in the midline of the rat's back (pay attention to heat preservation to avoid solidification of agar, and do not leak liquid to affect the weight of agar when pulling out the needle). Administration was started on the second day after modelling, once daily, for fifteen consecutive days. On the fifteenth day, the granuloma agar block was dissected and absorbed with a filter paper. The difference of granuloma weight between the administration group and the model group was compared to calculate the granuloma inhibition rate: granulomatous inhibition rate = (granuloma weight of model group − granuloma weight of administration group)/granuloma weight of model group × 100%.

### 2.3. Collection of Compounds in RSTF

#### 2.3.1. Data Preparation and Screening of Active Compounds

Composite compounds of each herb in RSTF were obtained from TCMSP (http://lsp.nwsuaf.edu.cn/tcmsp.php). TCMSP is a unique system pharmacology platform of Chinese herbal medicines that captures the relationships between drugs, targets, and diseases. The database includes chemicals, targets and drug-target networks, and associated drug-target-disease networks, as well as pharmacokinetic properties for natural compounds involving oral bioavailability, drug-likeness, intestinal epithelial permeability, blood-brain barrier, and aqueous solubility. Active compounds were selected by setting oral bioavailability (OB) ≥ 30%, drug-likeness (DL) ≥ 0.18, Caco-2 permeability (Caco‐2) ≥ ‐0.4, and drug half-life (HL) ≥ 4 as the threshold.

#### 2.3.2. Prediction of Drug Targets for RSTF

The prediction of drug targets was performed as previously described. The in silico prediction models, SEA search tool (SEArch, http://sea.bkslab.org/), and STITCH 4.0 (Search Tool for Interacting Chemicals, http://stitch.embl.de/) were combined to predict the target profiles of active herbal compounds.

#### 2.3.3. Collection of Targets Related to Pharyngitis

Pharyngitis-related targets were obtained from two main sources: differentially expressed genes (DEGs) obtained from publicly available microarray data and disease-related databases. To identify the main DEGs between normal and pharyngitis-related specimens, microarray data GEO34205, GEO17732, and GEO20262 were gained from the Gene Expression Omnibus database (GEO, https://www.ncbi.nlm.nih.gov/geo/), and 2000 differential genes were analyzed by each microarray.

#### 2.3.4. CPPI Network Construction

The putative RSTF-related target network and pharyngitis-related target network were constructed based on their interaction data. PPI data were imported from six currently available PPI databases, including the Biological General Repository for Interaction Datasets (BioGRID), the Biomolecular Interaction Network Database (BIND), the Molecular INTeraction Database (MINT), the Human Protein Reference Database (HPRD), and the Database of Interacting Proteins (DIP), searched by BisoGenet, a Cytoscape plugin. These two PPI networks were then merged to gain a core protein-protein interaction (CPPI) network by employing Cytoscape software (version 3.2.1).

#### 2.3.5. Enrichment and Pathway Analysis

The gene ontology (GO) analysis of RSTF-related targets was based on ClueGO which is a plugin for visualization of nonredundant biological terms for large gene clusters in a functionally grouped network. The ClueGO network was created by using kappa statistics, reflecting the relationships between the terms on the basis of the similarity between their associated genes. The significances of the terms and groups were calculated automatically. And the enrichment and pathway analysis based on DAVID (https://david.ncifcrf.gov/home.jsp) were used to perform the gene ontology (GO) or KEGG pathway enrichment analysis of the putative Pharyngitis-related targets and intersection of CPPI networks.

### 2.4. Antibacterial Effect of RSTF

#### 2.4.1. In Vitro

*Tube method*: take strain nutrition broth culture of bacteria cultured for eighteen hours, including standard Staphylococcus aureus, clinically isolated pneumococcus, Staphylococcus aureus, Pseudomonas cepacia, Staphylococcus epidermidis, and Beta streptococcus. Dilute at the concentration of 10^−6^ with sterilized physiological saline for experiments. Take ten sterilized test tubes; 1 mL of nutrient broth was added into each tube, and 1 mL of pharmaceuticals was added into the first tube. Mix the first tube, and take 1 mL liquid to the second tube, diluted in sequence. Discard 1 mL liquid of the ninth tube, and set the tenth tube as the control; no pharmaceuticals were added to it (Lanqin oral liquid was used as a positive control). Each tube was added with 0.1 mL of bacteria and cultured at 37°C for eighteen hours. Then inoculated loops were placed onto the nutrient agar plates with a loop from two adjacent bacteria and sterile growing test tubes. Bacterial growth was observed after eighteen-hour incubation at 37°C. The highest drug dilution with no bacterial growth was set as the minimum inhibitory concentration (MIC).

*Plate method*: take the broth agar medium and dissolve it and pour about 20 mL into a nine-centimeter plate until it solidifies. 0.1 mL of the bacteria solution was sucked with a 1 mL sterile syringe to evenly spread on solidified broth agar plates. After several minutes of drying, use a sterile steel ring to drill four holes. Inject 0.1 mL of pharmaceuticals into each plate, and apply one test bacteria to each plate. Two different liquids were injected: RSTF and Lanqin oral liquid. The diameter of the zone of inhibition was measured with a ruler after incubation at 37°C for twenty hours.

#### 2.4.2. In Vivo

Pretest the lethality of bacterial (Staphylococcus aureus and Beta streptococcus) infection in mice: different concentrations of the bacteria solution were injected into the abdominal cavity of the mice. Immediately observe the activity and the death time of mice after injection; the concentration of bacteria solution resulting in mortality at 80-90% within 72 hours was selected for formal experiments.

*Formal experiments*: mice weighing 18 g-22 g were randomly divided into seven groups of twenty in each group. Each group was intragastrically administered once daily for seven consecutive days. On the fifth day, each group of mice (except the normal control group) was intraperitoneally injected with 0.5 mL of the bacterial solution one hour after administration. The administration was continued for another two days. The deaths within seven days were observed to calculate survival days and survival rates.

### 2.5. Mouse Phagocytic Index

Mice weighing 18 g-22 g were randomly divided into five groups of ten in each group. Animals in each group were continuously gavage until 7 days, and Indian ink diluted at 1 : 5 with saline was injected into the tail vein (0.1 mL/10 g). Then take 20 *μ*L blood from the venous plexus, then immediately add it to 2 mL 0.1% Na2CO3 solution after 2 min (t1) and 10 min (t2) of the injection. The optical density (OD) at 600 nm was measured with a spectrophotometer. Mice were euthanized and dissected; the liver and spleen were taken out to weigh after the blood was blotted dry with a filter paper: *K* = (lgOD1 − lgOD2)/(t2 − t1); phagocytic index = body weight × *K* 1/3/(liver weight + spleen weight), where *K* is the clearance index; t2 is the second time to take the blood after the ink (10 min); t1 is the first time to take the blood after the ink (2 min); OD1 is the optical density of t1; and OD2 is the optical density of t2.

### 2.6. Proliferation and Transformation of Mouse Spleen Lymphocytes

The spleen cell suspension (5 × 10^5^/mL) was inoculated on a 96-well cell culture plate. After adding 200 *μ*L cell suspension to each well, groups were processed according to the experimental design. Each sample had six replicate wells. After culturing for 24, 48, and 72 hours, centrifugation was performed at 1000 r/min, and the supernatant was carefully discarded (excluding the colour interference of the drug itself). Add 90 *μ*L of 1640 medium per well and 10 *μ*L of MTT solution, and incubate for four hours. Centrifuge at 1000 r/min to remove supernatant, add DMSO to dissolve by shaking, and measure OD at 490 nm. Stimulation index (SI) is equal to the OD of a group treated by Con A/OD of control.

### 2.7. Determination of Serum Haemolysin

Three hours after the first administration, mice in the administration group were immunized by intraperitoneal injection of SRBC 0.2 mL (containing about 4 × 10^8^ SRBCs). Two hours after the last dose on the seventh day of immunization, blood samples were collected from the eye of mice. The blood was then kept at room temperature for one hour and subsequently centrifuged at 3000 rpm for 10 min at 4°C. One milliliter of diluted serum and 0.5 mL of 10% SRBC were added to the reaction tube. After 1 mL of complement was added to the tube (or 1 mL of 1 : 10 diluted guinea pig serum), the tube was placed at 37°C to incubate for ten minutes and then moved to an ice bath to stop the reaction. Centrifuge the tube at 2000 rpm for ten minutes, take 1 mL of the supernatant, and add 3 mL of Dulbecco's reagent (or distilled water) to mix. After ten minutes, mix each sample and pipet 200 *μ*L into a 96-well plate, and measure OD at 540 nm. The determination of serum haemolysin was expressed as the half value of haemolysis (HC50).

### 2.8. Leukocyte Migration

Each group of rats was administered prophylactically. After the seventh day of administration, each group of rats was anesthetized with ether and the back was epilated. Under aseptic conditions, inject 5 mL air to subcutaneously in the intrascapular region form a circular air sac. On the next day, 5 mL of 2% carboxymethyl cellulose (CMC) physiological saline solution was injected into the circular air sac of rats at 0.5 to 1 hour after the last administration. Then, 0.1 mL of liquid was drawn from the circular air sac at 3 and 7.5 hours, respectively. While collecting the liquid, shake the rat or gently press around the circular air sac to make the liquid well mixed. The number of white blood cells in the circular air sac was counted by a blood cell counter.

### 2.9. Western Blotting

The protein concentrations of the samples were determined using a BCA protein assay kit. Total protein extracts were resolved by 10% SDS-PAGE and transferred onto polyvinylidene difluoride membranes. After blocking with BSA, the membranes were washed five times for five minutes with Tris-buffered saline, containing 0.1% Tween-20 (TBST) and then incubated with antibodies against NF-*κ*B, p-NF-*κ*B, or GAPDH at 4°C overnight. After washing, membranes were incubated at room temperature with secondary peroxidase-linked goat anti-rabbit IgG for one hour. After washing, protein bands were detected by enhanced chemiluminescence, and the protein expressions were quantified by ChemiScope analysis.

### 2.10. Cell Culture

The NP69 cell line was provided by Berke Biology, Ltd. Cells were maintained in 1640 containing 10% FBS, streptomycin (100 *μ*g/mL), and penicillin (100 *μ*g/mL) and cultured at 37°C under 5% CO_2_.

### 2.11. Real-Time PCR

Total RNA was extracted from NP69 cells using an RNase mini kit (Transgen, Beijing, China) and reverse transcribed. Primers of IL-6, IL-8, IL-1*β*, and ICAM1 were designed and synthesized based on published sequences: IL-6 (5′-GAA GAG CGC CGC TGA GAAT-3′; 5′-GTG CAG AGG GTT TAA TGT CAA CT-3′); IL-8 (5′-TTT TGC CAA GGA GTG CTA AAGA-3′; 5′-AAC CCT CTG CAC CCA GTT TTC-3′); IL-1*β* (5′-ATG ATG GCT TAT TAC AGT GGC AA-3′; 5′-GTC GGA GAT TCG TAG CTG GA-3′); and ICAM-1 (5′-TTG GGC ATA GAG ACC CCG TT-3′; 5′-GCA CAT TGC TCA GTT CAT ACA CC-3′). Real-time PCR was performed using SYBR Green PCR Master Mix (Transgen, Beijing, China) and a 7500 Real-time PCR System (Thermo Fisher Scientific, New York, USA) according to the manufacturer's protocol.

### 2.12. Immunofluorescence Assay In Vitro

Cells were fixed with 4% paraformaldehyde for 15 min and washed with PBS for three times, 10 minutes each time. Cells were permeabilized with 0.5% triton X-100 for 10 min and washed with PBS for three times, 10 minutes each time. The cells were probed with 4 mg/mL rabbit monoclonal antibody directed against NF-*κ*B p65 at 4°C overnight. After repeated washes with PBS, the cells were probed with 1 mg/mL Rho-conjugated goat anti-rabbit IgG antibody for 1 h and 5 mg/mL 4′,6-diamidino-2-phenylindole (DAPI) for 10 min. The labelled sections were viewed with a fluorescence microscope (Zeiss).

### 2.13. Animal Anaesthesia and Euthanasia

All animals were maintained under specific pathogen-free conditions and handled in accordance with the Committee on Laboratory Animal Care of Nanjing University of Chinese Medicine; all animals were given humane care according to the guidelines of the National Institutes of Health. In the in vivo experiment, rats were anaesthetized with isoflurane during the surgical process and body temperature was kept using a warm blanket. Rats were euthanized by primary inhalation of isoflurane and secondary to cervical dislocation according to the AVMA Guidelines on Euthanasia.

### 2.14. Statistical Analysis

All data were expressed as the percentages and means with standard deviations (mean ± SD). Statistical analysis and plotting were conducted using Student's *t*-test and one-way ANOVA by GraphPad Prism 5 for Windows. *p* < 0.05 was considered statistically significant.

## 3. Results

### 3.1. RSTF Significantly Ameliorate the Symptoms of Pharyngitis

**Figure 1:**
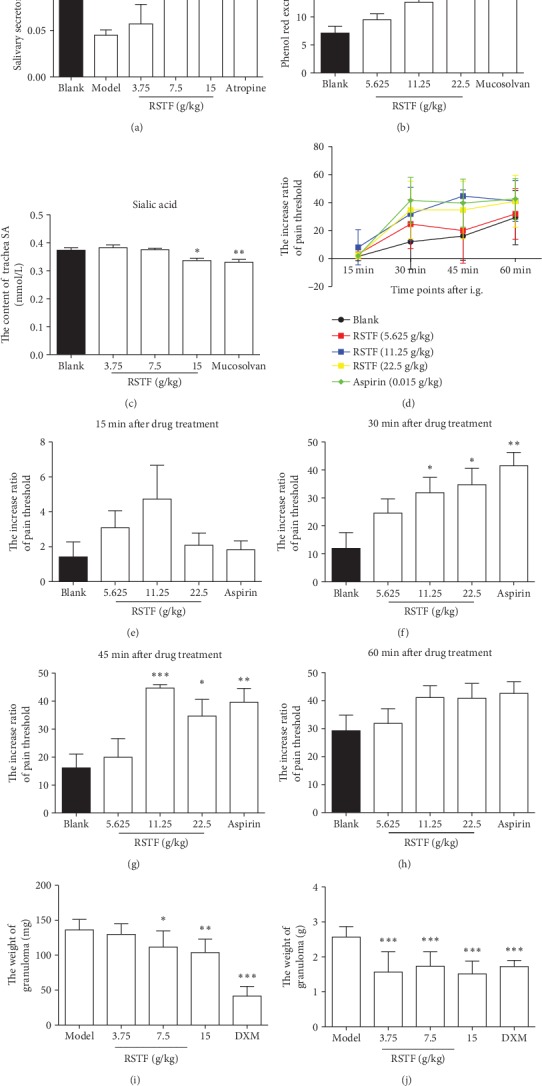
RSTF improves the symptoms of pharyngitis. (a) Effect of RSTF on saliva secretion in rats. (b) Effect of RSTF on excretion of phenol red in mice. (c) Effect of RSTF on sialic acid content in rat tracheal secretions. (d) Evaluation of the analgesic effect of RSTF by the hot plate method. (e–h) The increase ratio of pain threshold at 15, 30, 45, and 60 min after drug treatment. (i) The weight of cotton ball granuloma in rats. (j) The weight of agar-induced granuloma in rats. Data are presented as the mean ± SD. ^∗^*p* < 0.05, ^∗∗^*p* < 0.01, and ^∗∗∗^*p* < 0.001 versus the blank or model group.

Patients with pharyngitis are mainly characterized by dryness of the pharyngeal cavity, foreign body sensation, itching sensation, irritating cough, and aching feeling and swelling in the throat [19]. TCM usually depends on the principle of “Shengjin Runzao Huatan” to relieve symptoms of patients with pharyngitis. In our experiments, the rat model of dry mouth was established to evaluate the effects of “Shengjin Runzao Huatan” of RSTF. The results showed that RSTF boosted saliva secretion in rats at the concentration of 7.5 g/kg and 15 g/kg, and its promotion was slightly lower than that of the positive drug atropine (Figure 1(a)). This indicated that RSTF can effectively relieve the xerostomia symptoms in patients with pharyngitis. Can it effectively remove phlegm and thereby improve the symptoms of foreign body sensation, itching sensation, and irritating cough? A mouse phenol red excretion experiment was used to determine the eliminating phlegm effects of RSTF. The sialic acid in the sputum can increase the viscosity of the sputum, and the expectorant effect of RSTF can be further evaluated by detecting the sialic acid content in the rat tracheal secretions. The phenol red excretion results indicated that RSTF can significantly stimulate the excretion of sputum in mice at the concentration of 11.25 g/kg and 22.5 g/kg (Figure 1(b)). The results of sialic acid secretion showed that the positive drug ambroxol hydrochloride can significantly reduce the content of sialic acid in the trachea and sputum viscosity. The low dose of RSTF (3.75 g/kg) had no effect; when it reached 15 g/kg, the content of sialic acid in the rat tracheal was significantly reduced compared with the blank group (Figure 1(c)), indicating that RSTF has a certain effect on reducing the viscosity of phlegm.

Patients with pharyngitis are also accompanied by burning pain in the throat; therefore, we examined the analgesic effects of RSTF by using the hot plate method. Both RSTF and aspirin did not significantly increase the pain threshold of the mice at 15 minutes after the administration compared with the control group. The pain threshold at 30 minutes after administration at the dose of 22.5 g/kg was significantly higher than the control group; its analgesic effect is comparable to aspirin. The RSTF of 11.25 g/kg did not exert an analgesic effect until 45 minutes post administration, and the effect of the high dose of RSTF (22.5 g/kg) decreased, but aspirin still maintained a significant analgesic effect at this time. Only the high dose of RSTF still has a significant analgesic effect lasting to 60 minutes post administration (Figures 1(d)–1(h)). Pharyngitis often accompanied by throat inflammation and swelling; thus, two models were used to determine the anti-inflammatory and detumescence effects of RSTF. In the rat granuloma model induced by cotton ball implantation, the weight of granuloma in groups treated with RSTF at the concentration of 7.5 g/kg and 15 g/kg was lower than that of the model group, and the anti-inflammatory effect of Dexamethasone acetate (DXM) is the most significant (Figure 1(i)). RSTF also has a significant inhibitory effect on agar-induced proliferation of inflammatory granuloma in rats, and the effect is comparable to DXM (Figure 1(j)). The above results demonstrate that RSTF can significantly relieve the symptoms of patients with pharyngitis, which is quite important for improving the quality of life and mental status of patients.

### 3.2. RSTF Active Compound Screening

RSTF is effective in treating pharyngitis clinically. The experimental results also showed that RSTF can effectively relieve the symptoms of pharyngitis, but what is the pharmacological mechanism? The therapeutic mechanism of TCM has been difficult to clarify at the molecular biology level for a long period of time because of the complex chemical composition. There is still no valid tactic to identify the active compounds in medicinal herbs and legacy approach to detect the ingredients with an extremely low concentration.

**Figure 2:**
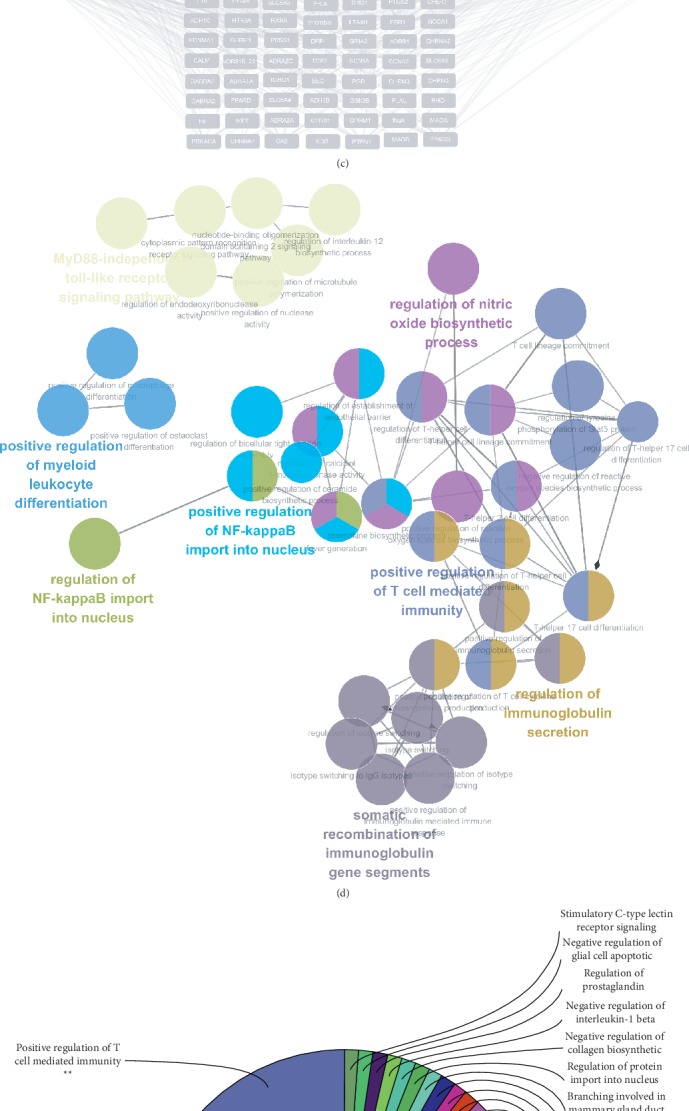
Screening and network construction of active ingredients. (a) Active ingredients in RSTF were screened for four ADME/Tox parameters. In total, 70 active compounds met the screening criteria. (b) ADME parameter distribution for different herbs. (c) Construction of the RSTF compound-putative target network. The nodes representing candidate compounds are shown as polychrome triangles (red: HMM; orange: FLL; yellow: AMK; green: CR; blue: PCW; cyan: OS; violet: EHH), and the targets are indicated by grey squares. (d, e) Candidate target identification and ClueGO pathway analysis. A functionally grouped network of enriched categories was generated for the target genes. GO terms are represented as nodes, and node size represents the term's enrichment significance. Functionally related groups partially overlap. Only the most significant term in the group was labelled. Representative enriched pathway (*p* < 0.05) interactions among the main RSTF targets.

Composite compounds of each herb in RSTF were obtained from TCMSP [20]. There is 174, 238, 110, 444, 68, 176, and 158 compound information, respectively, from HMM, FLL, AMK, CR, PCW, OS, and EHH in RSTF. The ADME model and literature confirmation were used to screen the active compounds of RSTF. We tested ADME properties of these ingredients including drug-likeness (DL), oral bioavailability (OB), Caco-2 permeability (Caco-2), and drug half-life (HL) based on published findings [21]. The recommended screening criteria were as follows: DL ≥ 0.18, OB ≥ 30%, Caco − 2 ≥ ‐0.4, and HL ≥ 4 [22]. At last, seventy potential compounds, including six duplicate components, passed ADME screening (Figure 2(a)). Additionally, among the compounds collected from TCMSP, ~50.9% of the molecules (696/1368) are orally bioavailable, but only 27.3% (374/1368) have long half-life. Interestingly, over 70.2% of the compounds (960/1368) were easily absorbed by Caco-2 cell monolayers and approximately 45.2% (618/1368) had drug-like characteristics (Figure 2(b)). A total of 70 active compounds passed ADME screening: 14 for HMM, 9 for FLL, 5 for AMK, 10 for CR, 14 for PCW, 15 for OS, and 15 for EHH (Supplementary Table S1).

### 3.3. Compound-Target Network Construction and Analysis

TCM formula exerts its efficacy by acting on multiple targets and pathological processes under normal circumstances. We used a systemic approach to predict the potential targets of the collected active ingredients of RSTF. We used a systematic target prediction approach to predict RSTF's potential targets based on its active ingredients. A total of 355 potential targets were predicted for the 70 candidate compounds: 73 for HMM, 57 for FLL, 14 for AMK, 48 for CR, 35 for PCW, 71 for OS, and 57 for EHH; of these, 84 targets remained after deleting duplicate ones. Detailed target information is presented in Supplementary Table S2.

By acting on multiple targets, TCM formulas exhibit versatile biological and pharmacological activities. Studying the complicated interactions between TCM compounds and their targets at the systematic level may help us understand the mechanisms underlying TCM effects. We constructed a compound-target network based on the candidate RSTF compounds and their potential targets to clarify the complex interactions between them. A total of 118 nodes and 573 compound-target interactions are presented in this network (Figure 2(c) and Supplementary Table S3). Although the numbers of targets in each herb were different, they overlapped dramatically in the six herbs (Table 1). The analysis also revealed that quercetin, kaempferol, hederagenin, beta-sitosterol, luteolin, and naringenin are present in multiple herbs, which may have synergistic beneficial effects on patients (Table 1). Most of these ingredients have anti-inflammatory and antibacterial actions. Moreover, beta-sitosterol and naringenin are known to have antitussive effects. Quercetin and luteolin are the main ingredients of HMM, FLL, and EHH, i.e., herbs that have been widely used in China to treat pharyngitis. Kaempferol from HMM, FLL, and EHH has the anti-inflammatory, antibacterial, and antioxidant effects. ClueGO, a Cytoscape plugin which integrates Gene Ontology (GO) terms as well as KEGG/BioCarta pathways which can create a functionally organized GO/pathway term network, was next applied to further study the potential pathways involved in the candidate targets of RSTF [23]. ClueGO enrichment analysis shows that candidate targets of RSTF could mainly be assigned to the immune-related and NF-*κ*B-related signal pathways (Figures 2(d) and 2(e)). And it is generally known that the NF-*κ*B signalling pathway is closely related to inflammation, immunity, bacterial infection, and oxidative reactions [24–27].

### 3.4. Collection of Pharyngitis-Related Targets and Preliminary Gene Ontology Analysis

**Figure 3:**
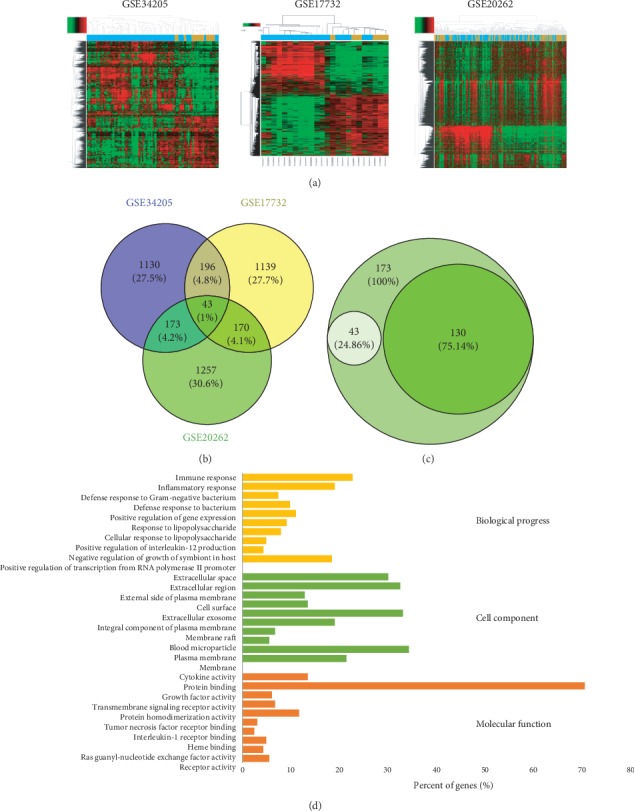
Collection of pharyngitis-related targets and preliminary gene ontology analysis. (a) 2000 differential genes of three GEO repository microarray data (GEO34205, GEO17732, and GEO20262) were analyzed, which were highly related to pharyngitis. Blue and brown stripes represent pharyngitis-related samples and normal samples. (b) 1542, 1548, and 1643 effective genes were chosen from three GEO repository microarray data; 43 mutual differential genes were screened. (c) 130 targets were obtained from PharmGKB, OMIM®, and DigSee; aggregate genes from GEO repository microarray data and another three databases and 173 pharyngitis-related targets were screened in all. (d) GO analysis for biological processes, cell component, and molecular function terms was performed on putative pharyngitis targets; the top 10 terms with *p* < 0.05 are shown. Those at the top are the most significant ones.

The corresponding targets of a disease determine its treatment in clinic; therefore, we collected pharyngitis-related targets from two main sources: differentially expressed genes (DEGs) obtained from publicly available microarray data and disease-related databases. As shown in Figure 3(a) and Figure S1, three GEO microarray data (GEO34205, GEO17732, and GEO20262) were chosen and 2000 differential genes of each microarray were analyzed. Among them, 1542, 1548, and 1643 effective genes were chosen. A total of 43 mutual differential genes were screened from the three GEO repository microarrays (Figure 3(b)), while other 130 targets were obtained from three databases: Pharmacogenomics Knowledge Base (PharmGKB) [28], the Online Mendelian Inheritance in Man (OMIM®) [29], and the Disease Gene Search Engine with Evidence Sentences (DigSee) [30]. Aggregate genes from GEO repository microarray data and another three databases and 173 pharyngitis-related targets were screened (Figure 3(c), Supplementary Table S4).

We used the QuickGO database which is based on DAVID [31, 32] to perform the gene ontology (GO) analysis of the putative pharyngitis-related targets by synthesizing the results of biological process (BP), cell component (CC), and molecular function (MF) terms [33]. In all, 334 BPs, 33 CCs, and 57 MFs that were enriched for this dataset were identified, of which 256 BPs, 25 CCs, and 39 MFs had *p* values < 0.05. Top ten remarkably enriched terms in the BP, CC, and MF categories are shown in Figure 3(d). The same categories of terms are ordered by *p* values with the cut-off set to 0.05, and those on the top are more significant. At the bottom of Figure 3(d), the percentage of genes involved in the term is presented. As shown in Figure 3(d), 22.70% of the targets are related to the immune response, while 19.02% are related to inflammatory response as revealed by BP analysis. The rest terms include defence response to bacteria, positive regulation of gene expression, and response to lipopolysaccharide. CC analysis showed that most identified proteins were distributed in the extracellular region, plasma membrane, blood microparticle, or membrane raft, and MF analysis showed 70.55% of the identified targets were shown to be binding proteins, while the rest terms are cytokine activity, protein homodimerization activity, and so on.

### 3.5. Identification of Candidate RSTF Targets for Pharyngitis Treatment

An increasing evidence from network biology shows that genes and proteins do not work independently, but rather work at multiple levels through interconnected molecular networks and pathways. Hence, we use proteins as nodes to establish network to clarify the mechanisms under the therapeutic effects of RSTF on pharyngitis. Protein-protein interaction (PPI) networks were established to reflect the properties of biological molecules. A putative target PPI network of RSTF (2116 nodes and 46543 edges) was firstly constructed by systematic pharmacology platform, and the PPI network of pharyngitis-related targets was constructed (4486 nodes and 116886 edges; Supplementary Figures S2-3) by multiple database retrieval results. These two networks were then merged to gain a core protein-protein interaction (CPPI) network that consisted of 1510 nodes and 39166 edges (Figure S4, Table S5).

**Figure 4:**
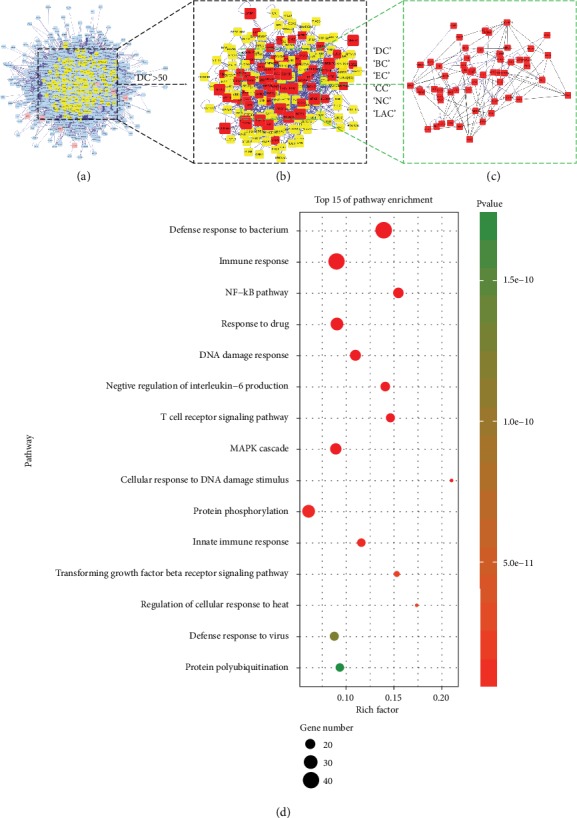
Identification of candidate RSTF targets for pharyngitis treatment. (a) Core protein-protein interaction (CPPI) network of RSTF targets. This network consists of 1510 nodes and 39166 edges. (b) Big hubs of the RSTF CPPI network extracted from (a) whose degree is more than twice the median degree of all nodes in the network. (c) PPI network of main RSTF targets extracted from (b) by calculating 6 topological features. (d) KEGG pathway enrichment analysis on the intersection of CPPI networks (*y*-axis label: represents the path name; *x*-axis label: rich factor; bubble area size: the number of genes in the target gene set that belongs to this pathway; bubble colour: enrichment significance, that is, the value of *p* value).

Therewith, candidate pharyngitis targets of RSTF were screened by using the topological features of CPPI. In the CPPI network, a node was identified as hub by filtering values greater than twice the median of all nodes in a network. Via a plugin of Cytoscape (CytoNCA), the main hubs of the network were screened by calculating topological features for each hub. The median values of “BC,” “DC,” “EC,” “CC,” “NC,” and “LAC” were 369.2803, 57, 0.011916, 0.457134, 10.08762, and 9.062261, respectively. Thus, 355 hubs with BC > 369.2803, DC > 57, EC > 0.011916, CC > 0.457134, NC > 10.08762, and LAC > 9.062261 were set as the main hubs. A flow chart of core target screening is presented in Figures 4(a)–4(c). Detailed topological features of the CPPI and 72 candidate targets are shown in Supplementary Table S5.

### 3.6. Pathway Enrichment Analysis for Candidate RSTF Targets

We performed KEGG pathway enrichment analysis on the intersection of CPPI networks and used the advanced bubble chart of OmicShare tools to visualize the enrichment results [34]. The result of the high-level bubble chart is a graph showing the gene status of enriched pathways through visual indicators such as value, colour, and graph size. The advanced bubble diagram is displayed in four dimensions: enriched content (pathway involved), enriched gene quantity, enrichment ratio (enrichment factor), and enriched salience. The principle of the bubble diagram is very simple; that is, according to the order of significance of enrichment from high to low, the first few pathways with the highest significance (generally 15-20) are selected, and the number of genes enriched is multidimensionally displayed. As shown in Figure 4(d), candidate targets could mainly be assigned to the defence response to the bacterium, immune response, NF-*κ*B pathway, response to drug, and DNA damage response signalling pathways. Of these, the first two pathways have well-established roles in inflammation-related diseases (such as pneumonia and sepsis) [35, 36]. Effective management of M. pneumoniae infections can usually be achieved with macrolides, tetracyclines, or fluoroquinolones by enhancing the body's immunity [37, 38]. Pharyngitis has the effect of local vascular congestion, inflammation of lymphoid tissue, and hyperplasia of fibrous connective tissue, which causes the response of the immune response, eventually resulting in recurrent refractory pharyngeal inflammation. Abnormal replication of bacterial DNA is also known to play a crucial role in chronic pharyngitis, which is also the candidate target of RSTF. NF-*κ*B is a nuclear transcription factor that regulates expression of a large number of genes that are critical for the regulation of apoptosis, viral replication, tumorigenesis, inflammation, and various autoimmune diseases [39].

### 3.7. RSTF Has Significant Antibacterial Activity In Vitro and In Vivo

As indicated by the system pharmacology prediction, a large number of candidate targets of RSTF were related to the bacteria process, which are closely related to pharyngitis. To confirm the antibacterial effect of RSTF, we observed the antibacterial activity of RSTF both in vivo and in vitro. The tube test showed that RSTF has good antibacterial activity against Staphylococcus aureus, Diplococcus pneumoniae, Staphylococcus aureus, Pseudomonas cepacia, Staphylococcus epidermidis, and Beta streptococcus. Bacteriostatic MIC range from 0.122 g/mL to 0.366 g/mL (Table 2). The results of the plate method show that RSTF has strong antibacterial activity against Staphylococcus aureus, Diplococcus pneumoniae, Staphylococcus aureus, Pseudomonas cepacia, Staphylococcus epidermidis, and Beta streptococcus. The diameter of inhibition zone ranged from 13.5 mm to 15 mm (Table 3).

**Figure 5:**
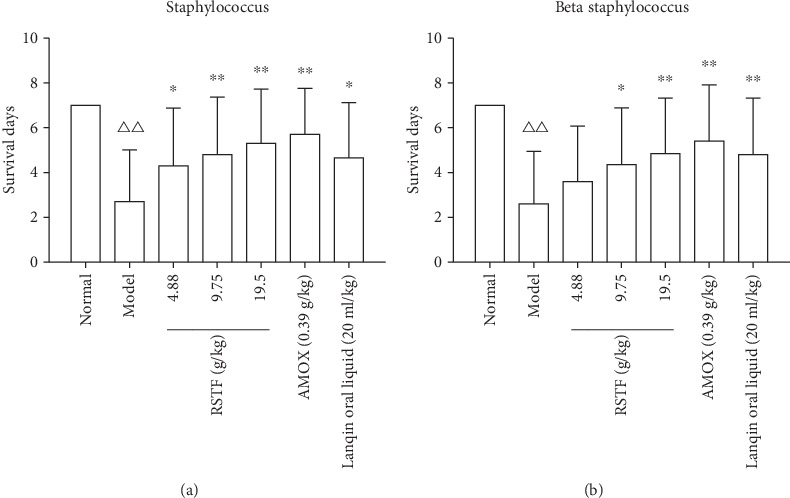
The antibacterial activity of RSTF in vitro and vivo. (a) Protective effects of RSTF on the death of mice infected with Staphylococcus aureus. (b) Protective effects of RSTF on the death of mice infected with Beta streptococcus (^△△^*p* < 0.01 compared with the normal group; ^∗^*p* < 0.05 and ^∗∗^*p* < 0.01 compared with the model group).

In order to further confirm the antibacterial effect of RSTF in vivo, we observed the protective effect of RSTF on mice infected with Staphylococcus aureus and Beta streptococcus. The low- (4.88 g/kg), medium- (9.75 g/kg), and high- (19.5 g/kg) dose intervention of RSTF on mice infected with Staphylococcus aureus can significantly prolong the survival time of mice and has a significant difference compared with the model group. The high- and medium-dose intervention of RSTF can significantly reduce the mortality of mice and increase the protection rate compared with untreated group (Figure 5(a)). Mice infected with Beta streptococcus intervention with the high- and medium-dose RSTF groups can significantly prolong the survival of mice compared with the untreated group. It can also reduce the deaths of animal and improve the protection rate (Figure 5(b)). The results above show that RSTF has a good antibacterial effect both in vitro and in vivo, indicating that RSTF can treat pharyngitis by directly killing bacteria.

### 3.8. The Effects of RSTF on Immunoregulation

**Figure 6:**
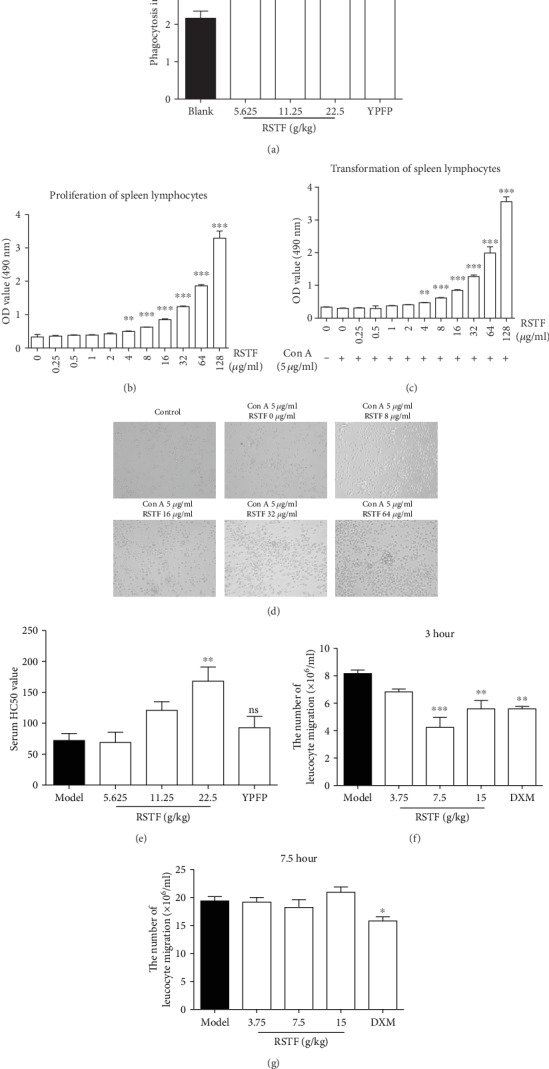
The effects of RSTF on immunoregulation. (a) Effect of RSTF on the mouse phagocytic index. (b) Effect of RSTF on proliferation of mouse spleen lymphocytes. (c) Effect of RSTF on transformation of mouse spleen lymphocytes. (d) Mouse spleen lymphocyte transformation at 48 h after drug treatment (×400). (e) Effect of RSTF on mouse serum haemolysin production. (f, g) Effect of RSTF on leukocyte migration in rat CMC sac at 3 h and 7.5 h after modelling. ^∗^*p* < 0.05, ^∗∗^*p* < 0.01, and ^∗∗∗^*p* < 0.001 versus the blank or model group.

From the above results, it is obvious that RSTF has a good antibacterial effect in vitro and in vivo. Combined with the results of enrichment analysis, we found that many targets of the intersection of RSTF and pharyngitis are related to immune regulation. Therefore, can RSTF enhance the body's immune system to produce antibacterial and pharyngitis and its complications? We conducted a carbon clearance test to detect the effect of RSTF on innate immunity of mice and monitored the proliferation and transformation of T and B lymphocytes, as well as the formation of haemolysin to verify the effect of RSTF on adaptive immunity of mice. The results of the carbon clearance test showed that the macrophage phagocytosis index in mice after RSTF treatment was significantly higher than that in the untreated group, indicating that RSTF can strengthen phagocytosis of macrophages in mice (Figure 6(a)). The proliferation of lymphocytes is one of the important indicators to reflect the body's immune function [40]. The spleen is an important immune organ in the body, rich in T and B lymphocytes and a small number of macrophages [41]. Lymphocytes in the spleen are easily separated and are important source of lymphocytes in vivo. There are nonspecific mitogen receptors on the surface of T lymphocytes, such as phytohemagglutinin (PHA) and concanavalin (ConA) [42, 43]. After being stimulated by mitogens in vitro or in vivo, they can be transformed into lymphoblastic cells. The T cell response function can be measured according to the degree of cell transformation [44]. Mouse spleen lymphocytes treated with RSTF in vitro significantly enhance the proliferation rate in a dose-dependent manner (Figure 6(b)). Direct treatment of mouse splenic lymphocytes with RSTF in vitro can significantly reinforce the conversion of spleen lymphocytes induced by ConA in a dose-dependent manner (Figure 6(c)). As shown in Figure 6(d), 48 hours after the treatment with ConA and RSTF at indicated concentration (0, 8 *μ*g/mL, 16 *μ*g/ml, 32 *μ*g/mL, or 64 *μ*g/mL), the size of lymphocytes increased significantly, the shape was irregular, the size of nuclei increased, and the nucleus osteoporosis was increased. The nucleolus was obvious, and the proportion of nuclear per cell decreased; meanwhile, some of the nuclei showed a dividing phase and the cytoplasm space became wider, and small bubble and pseudopodoids appeared at the rear of the cytoplasm (Figure 6(d)). The half haemolysis value of mice after the administration of RSTF was significantly increased compared with untreated group, and there was a significant difference. It indicated that RSTF can significantly intensify the formation of mouse haemolysin antibody and has the effect of enhancing specific immunity (Figure 6(e)). In Figures 6(f) and 6(g), it manifested that RSTF significantly inhibited leukocyte migration in rat saccharide cellulosic capsules at 3 h. RSTF exert the best inhibitory effect at the concentration of 7.5 g/kg. But the suppression effect disappeared at 7.5 h. This result demonstrated the inhibitory effect of RSTF on leukocyte migration occurs early in inflammation.

### 3.9. Regulatory Effect of RSTF on NF-*κ*B Signalling Pathway

**Figure 7:**
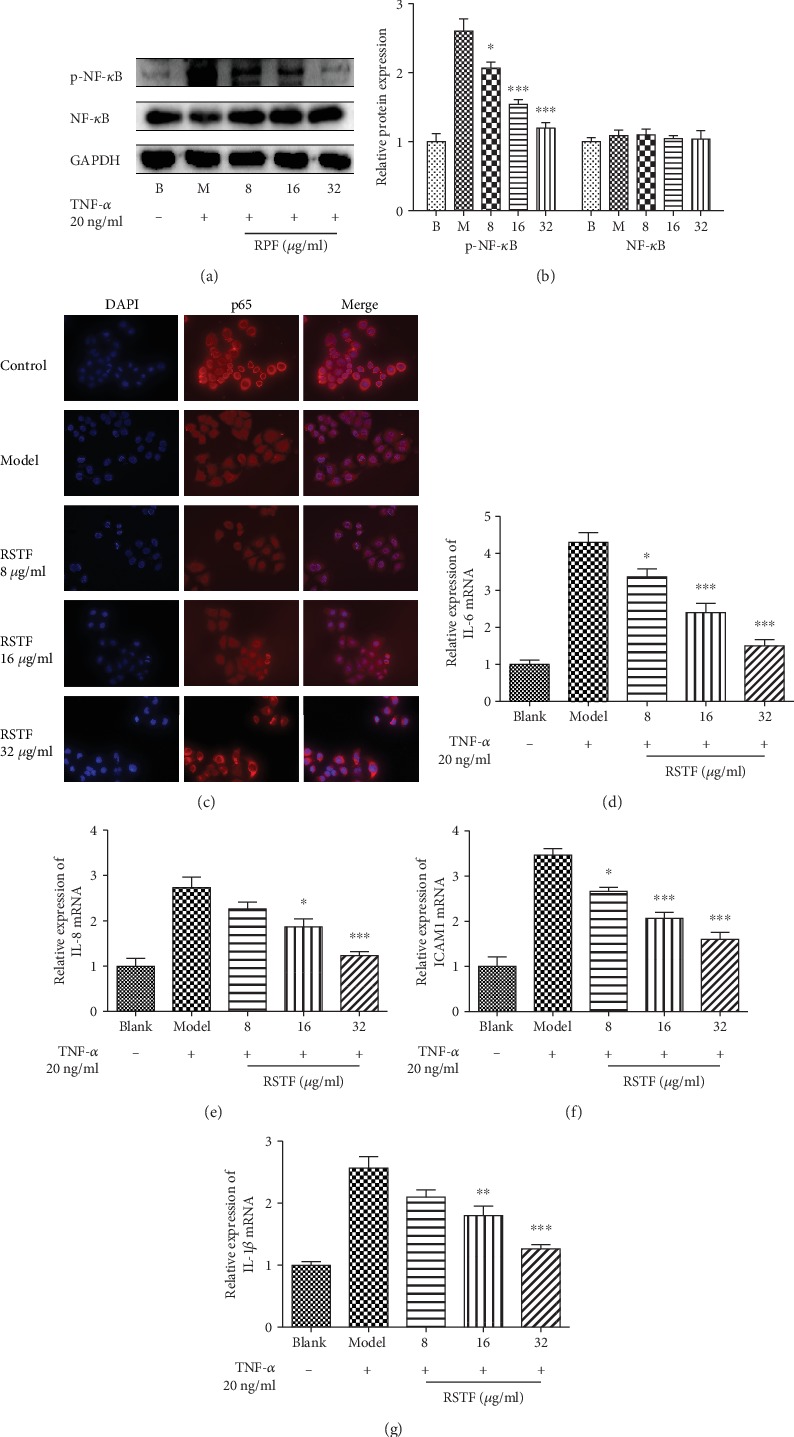
Regulatory effect on NF-*κ*B signalling pathway. (a) RSTF significantly inhibit the activation of NF-*κ*B induced by TNF-*α*. (b) Densitometric analysis of NF-*κ*B and p-NF-*κ*B expression. (c) RSTF inhibit NF-*κ*B p65 nuclear translocation in TNF-*α* induced NP69. (d–g) The mRNA expression of IL-1*β*, IL-6, IL-8, and ICAM1. ^∗^*p* < 0.05, ^∗∗^*p* < 0.01, and ^∗∗∗^*p* < 0.001 versus the model group.

From the pathway analysis of active ingredient of RSTF, it can be seen that RSTF mainly acts on the NF-*κ*B signalling pathway. After the coanalysis of the compound-target network and the disease-target network, the regulation effect of RSTF on the NF-*κ*B signalling pathway remains at the forefront. A large number of studies have shown that NF-*κ*B is inextricably linked with inflammation and immunity [39, 45, 46]. NF-*κ*B is a nuclear transcription factor which can be rapidly and transiently induced by viral and bacterial infections, necrotic cell products, DNA damage, oxidative stress, and proinflammatory cytokines [47–49]. If the activation of the NF-*κ*B signalling pathway cannot be resolved in time, it will lead to a series of inflammatory diseases such as rheumatoid arthritis, septic shock, and cancer [50–52]. Previous studies have shown that RSTF can enhance the body's specific immunity and nonspecific immunity. Could RSTF enhance the immune system by regulating the NF-*κ*B signalling pathway, thereby inhibiting the development of inflammation? Therefore, the normal nasopharyngeal epithelial cells NP69 was used to detect the effect of RSTF on NF-*κ*B signalling pathways. It was found that RSTF can significantly inhibit the activation of NF-*κ*B and show a dose-dependent manner (Figures 7(a) and 7(b)). It can also inhibit NF-*κ*B p65 nuclear translocation in TNF-*α* induced NP69 (Figure 7(c)).

Many molecules involved in the early stages of immune response and inflammatory response are regulated by NF-*κ*B, including TNF-*α*, IL-1*β*, IL-6, IL-8, and ICAM1 [53, 54]. Proteins that are induced by NF-*κ*B could in turn activate NF-*κ*B, which creates a vicious circle and expands the initial inflammatory response. The results suggested that the levels of IL-1*β*, IL-6, IL-8, and ICAM1 mRNA increased after being stimulated by TNF-*α* (20 ng/mL) in NP69 (Figures 7(d)–7(g)). But RSTF could significantly reduce the mRNA expression of IL-1*β*, IL-6, IL-8, and ICAM1 in a dose-dependent manner (Figures 7(d)–7(g)).

## 4. Discussion

RSTF is commonly used for pharyngitis in clinic which consisted of seven Chinese herbal medicines. Clinical treatment results show that RSTF has good therapeutic effects with little side effects. Our in vivo results further verified the therapeutic effects on relieving the symptoms of pharyngitis, which has significant antipyretic, analgesic, anti-inflammatory, and Shengjin Runzao Huatan effects. However, there is no relevant research on the mechanism of RSTF till now. TCM formula contains a variety of complicated constituents, which may produce additive effects on individual targets. Each target can also exert a synergistic effect at the same time; all of these together constitute its pharmacological mechanism. These features also make it difficult to explore the mechanism of TCM. Here, we use network pharmacology and system biology techniques to predict the onset mechanism of RSTF for pharyngitis. Subsequently, the results of the prediction are verified by further experiments.

In this article, we analyzed the possible mechanism of action of the TCM formula through the systemic and scientific interdisciplinary analytical method and verified its possible molecular mechanism through further experiment, rather than subjectively selecting one mechanism. First, we screened out the potent active compounds in RSTF based on pharmacokinetic parameters and predicted the targets of these active ingredients. In order to systematically analyze the interaction between RSTF's pharmacodynamic material components and potential targets, we constructed the compound-target network based on the candidate components selected and their corresponding potential targets. Then, we screened out pharyngitis-related disease targets by multiple disease target sites and three gene chip data to further establish a disease-target network. The compound-target network and disease-target network were used to perform protein-protein interactions, and corresponding protein interaction networks (PPIs) were constructed to obtain intersecting targets. Based on enrichment analysis of related targets, it was found that RSTF may play a role in the treatment of pharyngitis by participating in the regulation of the immune system, inhibiting the development of inflammation, and direct killing of bacteria. Using both in silico prediction and in vivo/in vitro analyses to explore the mechanism underlying TCM formula, we may find a breakthrough in traditional Chinese medicine research in the future.

## 5. Conclusions

The experimental results show that RSTF has a strong antibacterial effect in vivo and in vitro. In addition, RSTF also has a regulatory effect on the immune system, manifested in its ability on promoting the proliferation and transformation of splenic lymphocytes, as well as the effects of strengthening phagocytosis of macrophages in mice and the inhibiting leukocyte migration in rat saccharide cellulosic capsules. The results of cell experiments show that RSTF can inhibit NF-*κ*B activation mediated by TNF-*α* and inhibit its entry into the nucleus. The mRNA expressions of IL-1*β*, IL-6, IL-8, and ICAM1 regulated by NF-*κ*B are inhibited by RSTF in a dose-dependent manner. In summary, consistent with the analysis results, RSTF can directly antimicrobically regulate the innate immunity and inhibit the development of inflammation through the NF-KB signalling pathway, so as to achieve the effect of relieving pharyngitis symptoms.

## Figures and Tables

**Table 1:** Different herbs contain the same ingredients and have similar effects.

Components	Pharmacological activity	Herbs
Quercetin	Anti-inflammatory, antiasthmatic, antitussive, antioxidant, antibacterial, anticancer, protect the cardiovascular system	HMM, FLL, OS, EHH
Kaempferol	Anti-inflammatory, antibacterial, antioxidant, antihypertension	HMM, FLL, EHH
Hederagenin	Antidepressant, stimulate mucosal, antiatherosclerosis, anticancer	HMM, PCW
Beta-sitosterol	Antitussive, lower cholesterol, anticancer	FLL, CR, OS, EHH
Luteolin	Antioxidant, anti-inflammatory, neuroprotection, anticancer, antiallergy, antifibrosis	FLL, EHH
Naringenin	Anti-inflammatory, antibacterial, antioxidant, antihyperlipidemia, antitussive, antiasthmatic	Cr、EHH

**Table 2:** Antibacterial effect of RSTF in vitro (tube method, *n* = 2).

Strains	MIC	
	RSTF (0.975 g/mL)	Lanqin oral liquid (Stoste, equivalent to 1 g/mL)
*Staphylococcus aureus*	0.244	0.25
*Diplococcus pneumoniae*	0.183	0.125
*Staphylococcus aureus*	0.183	0.125
*Pseudomonas cepacia*	0.122	0.125
*Staphylococcus epidermidis*	0.366	0.125
*Beta streptococcus*	0.122	0.125

**Table 3:** Antibacterial effect of RSTF in vitro (plate method, *n* = 2).

Strains	The diameter of inhibition zone (mm)	
	RSTF (0.975 g/mL)	Lanqin oral liquid (Stoste, equivalent to 1 g/mL)
*Staphylococcus aureus*	0	0
*Diplococcus pneumoniae*	13.5	13.25
*Staphylococcus aureus*	15	15.25
*Pseudomonas cepacia*	14	14
*Staphylococcus epidermidis*	0	0
*Beta streptococcus*	13.75	12.5

## Data Availability

The data used to support the findings of this study are available from the corresponding author upon request.

## Supplementary Materials

Supplementary 1 Supplementary Figure 1: identification of pharyngitis-related targets by pre-existing microarray data. 2000 differential genes of three GEO repository microarray data (GEO34205, GEO17732, and GEO20262) were analyzed, which were highly related to pharyngitis. 1542, 1548, and 1643 effective genes were chosen from three GEO repository microarray data. Volcano plots show differentially expressed genes (red), whereas light blue dots are genes with no significantly different expression. Supplementary Figure 2: PPI network of putative RSTF targets. 2116 nodes and 46543 edges were identified. Supplementary Figure 3: PPI network of CKD-related targets. 4486 nodes and 116886 edges were identified. Supplementary Figure 4: core protein-protein interaction (CPPI) network. The CPPI network consisted of 1510 nodes and 39166 edges.

Supplementary 2 Supplementary Table 1. ADME parameters of active compounds in RSTF.

Supplementary 3 Supplementary Table 2. Information of predicted RSTF targets.

Supplementary 4 Supplementary Table 3. Compound-target interaction information.

Supplementary 5 Supplementary Table 4. Information of known pharyngitis-related targets.

Supplementary 6 Supplementary Table 5. PPI data of interaction network. Supplementary materials and methods. (1) Data preparation and screening of active compounds. Composite compounds of each herb in RSTF were obtained from TCMSP (http://lsp.nwsuaf.edu.cn/tcmsp.php). The database includes chemicals, targets and drug-target networks, and associated drug-target-disease networks, as well as pharmacokinetic properties for natural compounds involving oral bioavailability, drug-likeness, intestinal epithelial permeability, blood-brain barrier, and aqueous solubility. Active compounds were selected by setting OB ≥ 30%, DL ≥ 0.18, Caco‐2 ≥ ‐0.4, and HL ≥ 4 as the threshold. (2) Prediction of drug targets for RSTF. The prediction of drug targets was performed as previously described. The in silico prediction models, SEA search tool (SEArch, http://sea.bkslab.org/), and STITCH 4.0 (Search Tool for Interacting Chemicals, http://stitch.embl.de/) were combined to predict the target profiles of active herbal compounds. (3) Collection of targets related to pharyngitis. Pharyngitis-related targets were obtained from two main sources: differentially expressed genes (DEGs) obtained from publicly available microarray data and disease-related databases. To identify the main DEGs between normal and pharyngitis-related specimens, microarray data GEO34205, GEO17732, and GEO20262 was gained from the Gene Expression Omnibus database (GEO, https://www.ncbi.nlm.nih.gov/geo/), and 2000 differential genes were analyzed by each microarray. (4) CPPI network construction. The putative RSTF-related target network and pharyngitis-related target network were constructed based on their interaction data. PPI data were imported from six currently available PPI databases, including the Biological General Repository for Interaction Datasets (BioGRID), the Biomolecular Interaction Network Database (BIND), the Molecular INTeraction Database (MINT), the Human Protein Reference Database (HPRD), and the Database of Interacting Proteins (DIP), searched by BisoGenet, a Cytoscape plugin. These two PPI networks were then merged to gain a core protein-protein interaction (CPPI) network by employing Cytoscape software (version 3.2.1). (5) Enrichment and pathway analysis. The gene ontology (GO) analysis of RSTF-related targets was based on ClueGO which is a plugin for visualization of nonredundant biological terms for large gene clusters in a functionally grouped network. The ClueGO network was created by using kappa statistics, reflecting the relationships between the terms on the basis of the similarity between their associated genes. The significances of the terms and groups were calculated automatically. And the enrichment and pathway analysis based on DAVID (https://david.ncifcrf.gov/home.jsp) were used to perform the gene ontology (GO) or KEGG pathway enrichment analysis of the putative pharyngitis-related targets and intersection of CPPI networks.
