# Supplementary material for: Relieving Sore Throat Formula Exerts a Therapeutic Effect on Pharyngitis through Immunoregulation and NF-κB Pathway
Source: Mediators Inflamm. 2020 May 15;2020:2929163. doi: 10.1155/2020/2929163 (PMC7245656; doi:10.1155/2020/2929163)
Supplement: Supplementary 1 — Supplementary Figure 1: identification of pharyngitis-related targets by pre-existing microarray data. 2000 differential genes of three GEO repository microarray data (GEO34205, GEO17732, and GEO20262) were analyzed, which were highly related to pharyngitis. 1542, 1548, and 1643 effective genes were chosen from three GEO repository microarray data. Volcano plots show differentially expressed genes (red), whereas light blue dots are genes with no significantly different expression. Supplementary Figure 2: PPI network of putative RSTF targets. 2116 nodes and 46543 edges were identified. Supplementary Figure 3: PPI network of CKD-related targets. 4486 nodes and 116886 edges were identified. Supplementary Figure 4: core protein-protein interaction (CPPI) network. The CPPI network consisted of 1510 nodes and 39166 edges. [file 2929163.f1.docx]

**GSE34205**


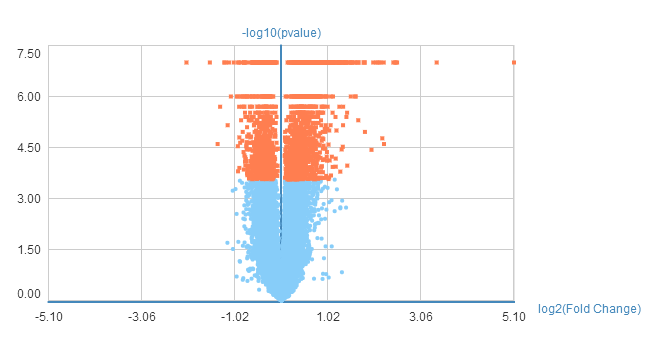


**GSE17732**


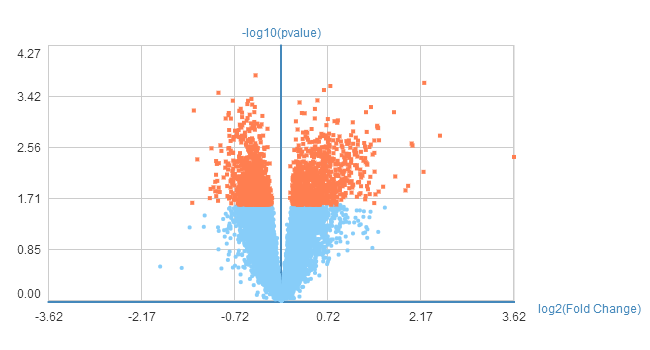


**GSE20262**


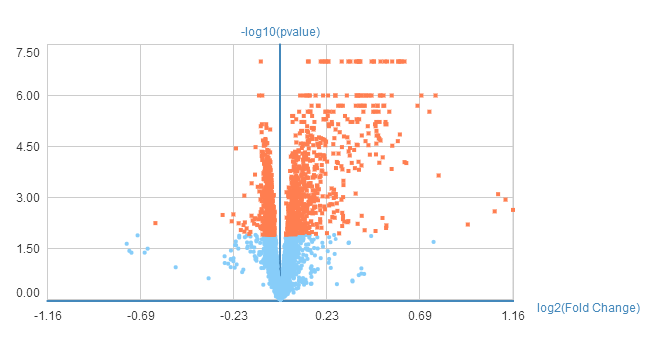


Supplementary Figure 1: Identification of pharyngitis-related targets by pre-existing microarray data. 2000 differential genes of three GEO repository microarray data (GEO34205、GEO17732 and GEO20262) were analysied, which were highly related to pharyngitis. 1542、1548 and 1643 effective genes were chosen from three GEO repository microarray data. Volcano plots show differentially expressed genes (red), whereas light blue dots are genes with no significantly different expression.


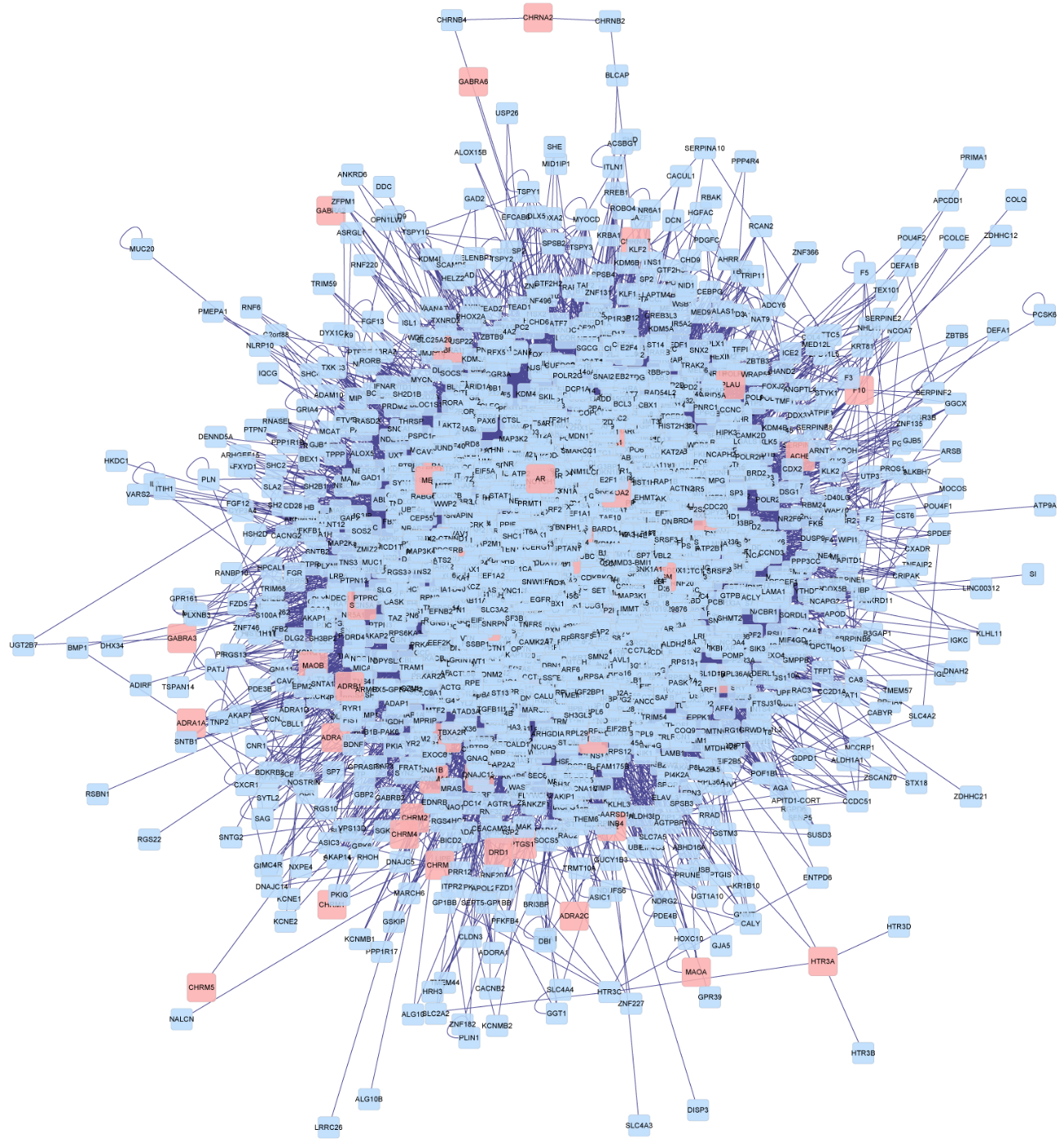


**Supplementary Figure 2:** PPI network of putative RSTF targets. 2116 nodes and 46543 edges were identified.


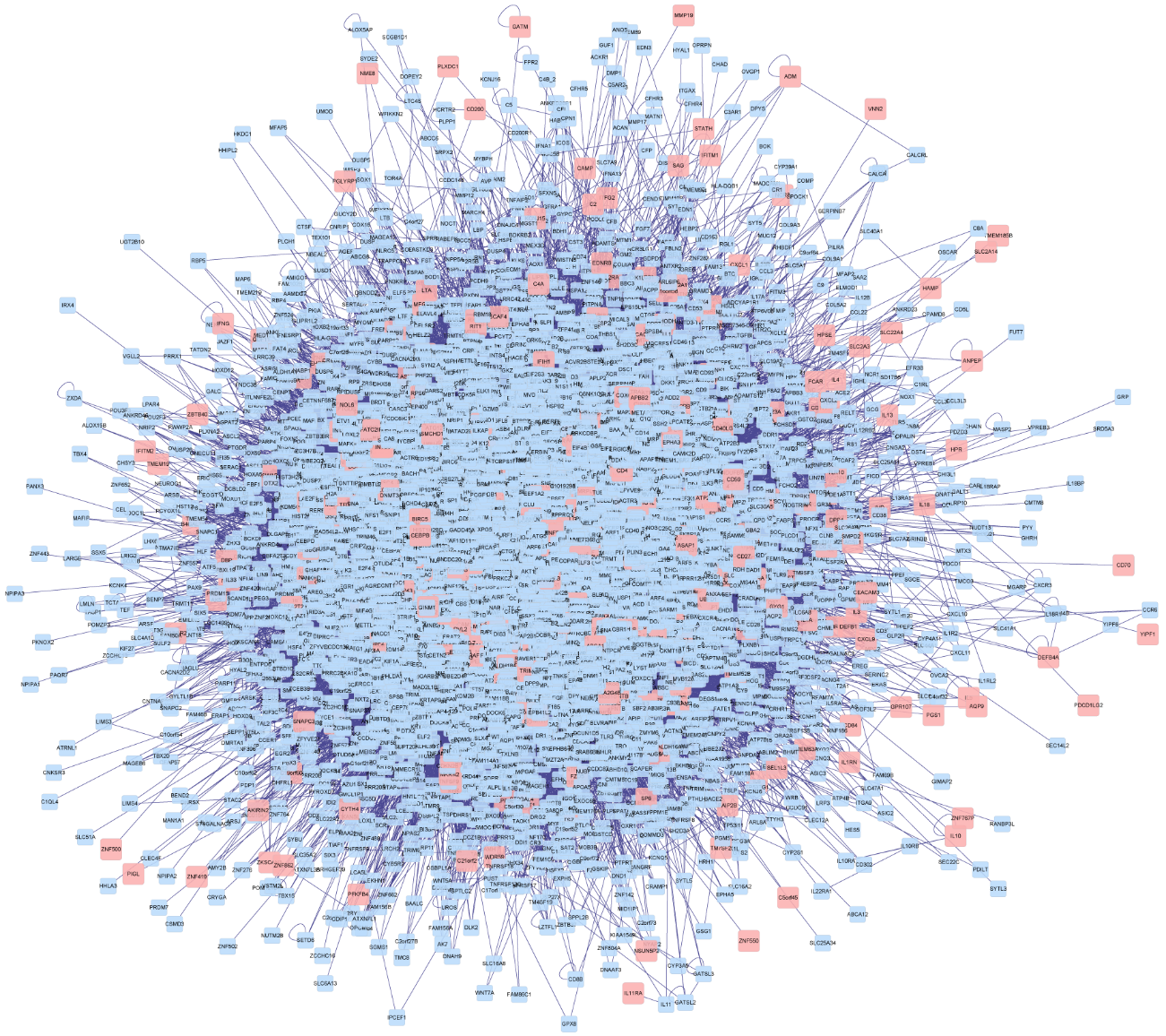


**Supplementary Figure 3:** PPI network of CKD-related targets. 4486 nodes and 116886 edges were identified.


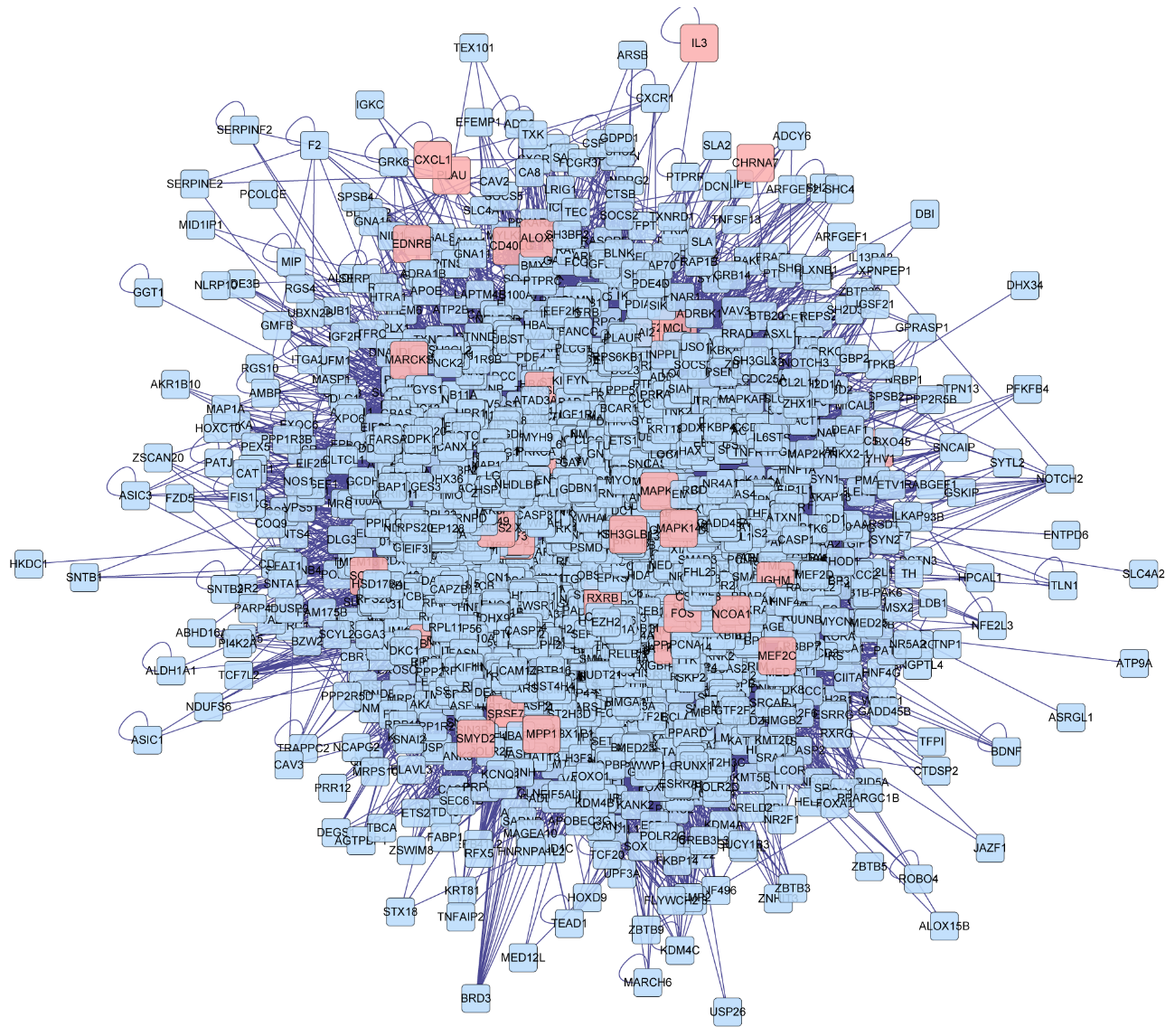


**Supplementary Figure 4:** Core protein-protein interaction (CPPI) network. The CPPI network consisted of 1510 nodes and 39166 edges.
